# Effectiveness of e-learning to promote oral health education: A systematic review and meta-analysis

**DOI:** 10.1097/MD.0000000000036550

**Published:** 2023-12-22

**Authors:** Yoshino Kaneyasu, Hideo Shigeishi, Masaru Sugiyama, Kouji Ohta

**Affiliations:** a Department of Public Oral Health, Program of Oral Health Sciences, Graduate School of Biomedical and Health Sciences, Hiroshima University, Hiroshima, Japan; b Department of Oral Health Sciences, Faculty of Health Care Sciences, Takarazuka University of Medical and Health Care, Takarazuka City, Hyogo, Japan.

**Keywords:** computer-assisted instruction, distance learning, e-learning, oral health education, randomized controlled trial

## Abstract

**Background:**

In recent times during and after the COVID-19 pandemic, e-learning is increasingly being used to give oral health education. However, the efficacy of e-learning in improving and promoting the oral hygiene and oral health knowledge, attitude and practice is unclear. Therefore, this systematic review and meta-analysis aim to clarify the effectiveness of e-learning compared to other conventional education methods for providing oral health.

**Methods:**

An electronic database search was performed on PubMed-Medline, Scopus, and CENTRAL (Central Register Cochrane of Controlled trials). Randomized controlled trials (RCTs), including cluster or group RCTs, were collected in this study. The risk of bias was assessed with the Cochrane Handbook for Systematic Reviews of Interventions. Five different meta-analyses were conducted for plaque index, gingival index, oral health knowledge, oral health attitude, and oral health practice using a random effects model.

**Results:**

A total of 282 articles were found through the database search; 19 articles were included in the qualitative synthesis and 9 articles in the quantitative synthesis. The meta-analysis found that compared with conventional education, e-learning exhibited no positive effect. However, the use of e-learning was superior to conventional education methods for oral health practice for adults in subgroup analysis.

**Conclusions:**

This paper could not indicate the effectiveness of e-learning in comparison with conventional education for oral health in total. However, for adults, it may be effective to get the oral health practice compared to the conventional education. Our study limitation is that there are only few studies that have assessed the effectiveness of e-learning. Therefore, numerous further high-quality studies should be conducted regarding the efficacy of e-learning compared with conventional education methods for oral health promotion.

## 1. Introduction

Oral health is vital for the maintenance one’s general health. Therefore, oral health education is conducted in a range of environments—such as schools, hospitals, and health care centers.^[1]^ In addition, oral health education provides information for promoting one oral health, such as improving oral health status, ensuring a healthy lifestyle, and breeding effective oral health attitudes.^[2]^ Dental health education for oral health promotion is crucial in dental health services and is delivered for individuals and groups in a dental setting, or for a large population through mass media.^[3,4]^ Until now, there have been various teaching methods regarding oral and dental health education, such as lectures; pamphlets,^[5–8]^ videos,^[5]^ DVDs,^[9,10]^ booklets,^[6,11,12]^ and new technologies on the internet, such as e-learning; mobile phones; and mobile applications (apps).^[13–20]^

Various methods of oral health education have been developed and advances in information and communication technology resulted in remarkable progress in recent years.^[21]^ This change has partly been caused by the COVID-19 pandemic, which affects the various aspects of human life, including education, health and medical care, and the economy.^[22]^ Therefore, a new educational culture, such as distance learning and non-face-to-face education, is widely been applied globally.

E-learning in the distance learning model has spread rapidly, being increasingly implemented during the COVID-19 pandemic.^[22,23]^ The main definition of e-learning implies the utilization of electronic means of communication for education, namely web-based and computer-based learning.^[24]^ The involvement of education using e-learning has been reported in the past several years.^[25–27]^ However, little is known about the effectiveness of e-learning education methods in comparison with those of conventional education, such as pamphlets, DVDs, and booklets, for oral health promotion, oral health knowledge, attitude, and practice (oral health KAP). To assess the effectiveness of e-learning is particularly significant for the need to help people who face difficulty going to the hospital by themselves or who live in less populated areas far from the hospital to improve their daily health because education using e-learning can be received anytime and anywhere. In addition, although face-to-face oral health education in schools is currently difficult due to the COVID-19 pandemic, the education by using e-learning education may built daily oral health habits for adolescents. Thus, we aimed to evaluate the effectiveness of e-learning on the internet compared with conventional education methods in improving and promoting oral hygiene status and oral health KAP.

## 2. Methods

This systematic review and meta-analysis followed the Preferred Reporting Items for Systematic Review and Meta-Analysis (PRISMA)^[28]^ and Cochrane guidelines.^[29]^ The following database search was performed: PubMed-Medline (January 1,2000 to January 1,2023-January 1,2000 to December 31,2022) : “e-learning” [Title/Abstract] OR “Computer-Assisted Instruction” [MeSH Terms] OR “distance learning” AND “health education, dental” [MeSH Terms] OR “oral health instructions” OR “oral health education” AND “randomized controlled trial” [Filter]. CENTRAL (Central Register Cochrane of Controlled Trials; January 1,2000 to December 31,2022) . Scopus (January 1,2000 to August 3,2023) ).

All the described databases were reviewed and assessed according to title, abstract, and full-text of each study by 2 independent reviewers. In case of disagreements, a third reviewer carefully assessed and resolved the matter. Furthermore, all data were entered into an Excel file (Microsoft, USA) for screening and recording eligibility criteria. Included and extracted data by the authors in this qualitative synthesis were the year of publication, country, duration of the study, number of subjects in the e-learning group/conventional or no intervention group, age (in years), and outcomes assessed by the original authors.

### 2.1. Eligibility criteria

The eligibility criteria of the participants, interventions, comparisons, outcomes, and study design in this qualitative and quantitative syntheses are as follows:

#### 2.1.1. Inclusion criteria.

Population: Adults, adolescents, and child; Interventions: E-learning, such as distance learning, online webinars, and oral health education by using mobile phone and mobile applications (apps); Comparison: Conventional education methods (e.g., face-to-face education and distribution of leaflets, booklets, CD-ROM, and DVD); Outcomes: Plaque index, Gingival index, Oral health knowledge, Oral health attitude, and Oral health practice (oral health KAP); Study design: Randomized controlled trials (RCTs), including cluster or group RCTs.

#### 2.1.2. Exclusion criteria.

The first exclusion criteria involved a systematic review, meta-analysis, study protocol, and study that did not include distance learning, having no connection to the internet, not being conducted in the English language, and not being relevant to oral health. In addition, studies that contained both groups being provided with e-learning, or a combination of e-learning with various conventional educational methods, were excluded from this qualitative synthesis. The second exclusion criteria involved no intervention in the control group means not continued oral health education, such as offered the only first intervention. In addition, outcomes are not including the plaque index, gingival index, oral health knowledge, oral health attitude, and oral health practice in this quantitative synthesis.

### 2.2. Risk bias assessment

The risk of bias in this study was assessed with the Cochrane Handbook for Systematic Reviews of Interventions and Review Manager 5.4.1 (Cochrane Collaboration, United Kingdom).^[29]^ This method applied the following 7 criteria: random sequence generation (selection bias); allocation concealment (selection bias); blinding for participants and personnel (performance bias); blinding for outcome assessors (detection bias); incomplete outcome data (attrition bias); selective reporting (reporting bias); and other bias.

### 2.3. Certainty assessment

We assessed the certainty of the evidence in the results about plaque index, gingival index, and oral health KAP by using the GRADEpro GDT software.^[30]^

### 2.4. Statistical analysis

Continuous data on mean difference and standard deviation were obtained from the selected 9 publications by the authors as follows: mean difference in plaque index (the baseline and end intervention) and standard deviations (SD); mean difference in the gingival index (the baseline and end intervention) and standard deviations (SD); mean difference in oral health knowledge (the baseline and end intervention) and standard deviations (SD); mean difference in oral health attitude (the baseline and end intervention) and standard deviations (SD); and mean difference in oral health practice (the baseline and end intervention) and standard deviations (SD). The SD of the difference was calculated using the formula reported by Sun et al^[31]^ The analysis of outcomes in this study was indicated by total and subgroups for developmental stages in the plaque index, gingival index, and the oral health KAP. A *P* value < .05 was considered significant. Besides, heterogeneity was assessed by the chi-square test and I^2^ statistics. The meta-analysis in this research with a random effects model was analyzed using Review Manager 5.4.1.

### 2.5. Ethical review

Since this research was systematic review and meta-analysis and did not address direct participants, ethical approval was not necessary.

## 3. Results

### 3.1. Search and selection results

**Figure 1.**
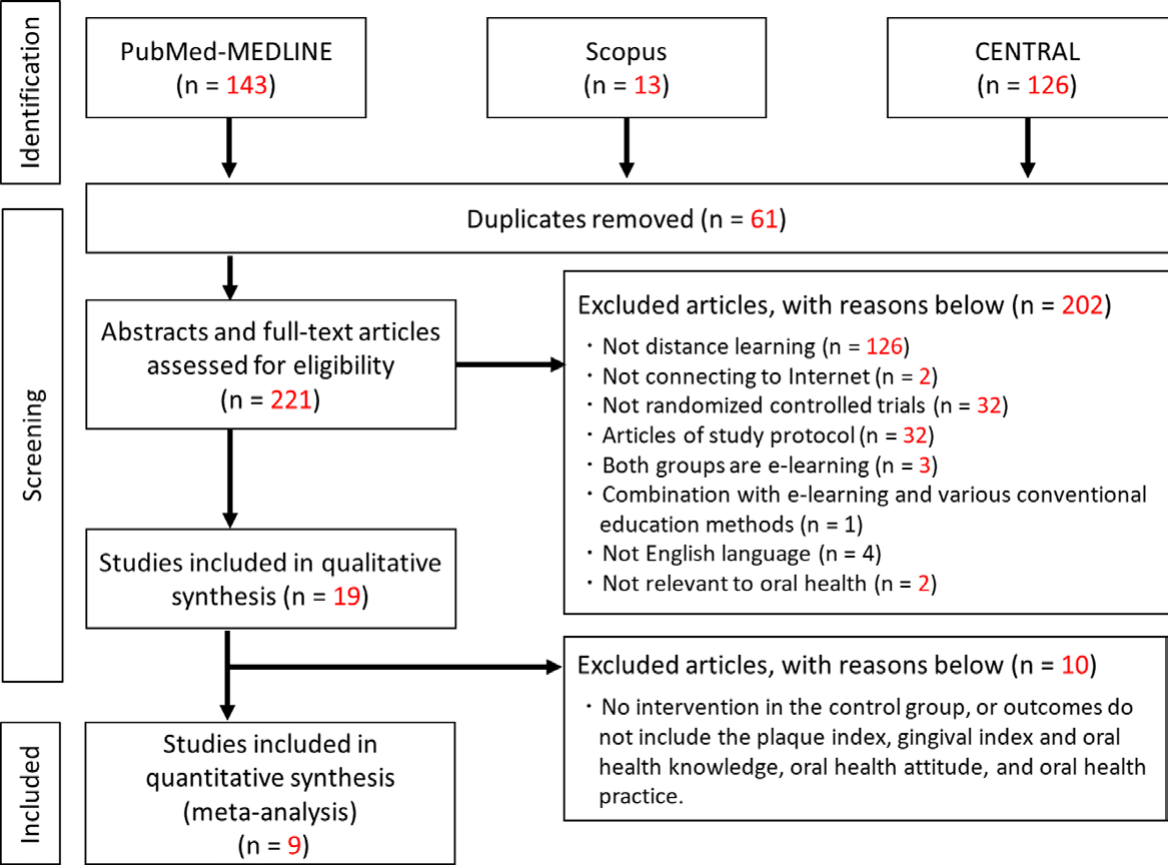
PRISMA flow-chart of the screened studies. PRISMA = Preferred Reporting Items for Systematic Review and Meta-Analysis.

Overall, 282 articles were found through the database search and removed 61 duplicated studies by screening titles and abstracts. Subsequently, 221 abstracts and full-text articles were assessed by the first exclusion criteria. Eventually, the review yielded 19 publications^[17,21,32–48]^ in qualitative synthesis (Table 1), and 9^[21,33,35,36,39,42,44,46,47]^ in quantitative synthesis by the second exclusion criteria. Table 1 indicated the characteristics of collected studies in qualitative synthesis. All the selected studies were published in English and implemented RCTs. The flow diagram of this study and exclusion reasons can be seen in Figure 1.

**Table 1:** Summary of the selected studies.

Author (yr), country	Duration of study	E-learning group	Conventional or no intervention group	Age (in yr)	Outcomes assessed
Lotto et al (2020), Brazil^[17]^	6 mo	Sending text messages via mobile phone: n = 31	No intervention: n = 52	E-learning group: 3.4 ± 0.6 No intervention group: 3.6 ± 0.6	VPI, ICDAS, eHEALS, and eating habits
Deokar et al (2021), India^[21]^	3 mo	Webinar: n = 260	Oral health education with PowerPoint: n = 261 No intervention: n = 270	E-learning group: 14.95 ± 0.79 Conventional group: 15.02 ± 0.81	OHI and GI
Ki et al (2021), South Korea^[32]^	6 wk	Mobile apps: n = 20	No intervention: n = 20	65 older	Tongue pressure, subjective oral dryness, USFR, and SWAL-QoL
Al-Ak’hali et al (2020), Saudi Arabia^[33]^	3 mo	Mobile apps and folio: n = 24	Folio: n = 19	E-learning group: 26.83 ± 5.27 Conventional group: 26.58 ± 4.72	PI and GI
Sarwer-Foner et al (2021), Brazil^[34]^	4 wk	Mobile apps: n = 40	No intervention: n = 37	E-learning group: 12.62 ± 1.20 No intervention group: 12.54 ± 1.66	VPI, GBI, DMFT, routine dental examinations, and toothbrushing and flossing frequency
Bonabi et al (2019), Iran^[35]^	4 mo	Mobile apps: n = 43	Booklet: n = 43	E-learning group: 39.22 ± 7.28 Conventional group: 44.26 ± 7.68	Oral health knowledge, attitude, and practice
Scheerman et al (2020), Netherlands^[36]^	12 wk	Mobile apps Clinical indices: n = 62 self-reported: n = 61	Oral health education at visiting treatment Clinical indices: n = 62 self-reported: n = 61	E-learning group: 13.2 ± 1.01 Conventional group: 13.5 ± 0.97	Al-Anezi and Harradine PI , BMOP, and oral health behaviors
Alkilzy et al (2019), Germany^[37]^	12 wk	Mobile apps: n = 26	No intervention: n = 23	5.1 ± 0.62	PBI and QHI
Marchetti et al (2018), Brazil^[38]^	20 wk	Mobile apps and oral education: n = 66 Mobile apps and video education: n = 63	No intervention (oral): n = 71 No intervention (video): n = 63	E-learning and video group: 15.98 ± 1.13 No intervention (video) group: 16.06 ± 1.2	OHI-S, GBI, and oral health knowledge
Al Bardaweel et al (2018), Syria^[39]^	12 wk	Video education program with the internet n = 100	Leaflet: n = 100	E-learning group: 10.8 ± 0.4 Conventional group: 10.69 ± 0.47	PI, GI, and oral health knowledge
Updegraff et al (2015), United States^[40]^	6 mo	Video with the internet (pmv): n = 278 Video with the internet (nmv): n = 288	No intervention: n = 128	E-learning group (pmv): 43.69 ± 14.81 E-learning group (nmv) 45.46 ± 15.8 No intervention group: 44.25 ± 15.84	Self-reported flossing, BIS, BAS, and self-efficacy
DeBate et al (2013), United States^[41]^	3 wk	Education program with the Internet: n = 349	No intervention: n = 259	18–41 older	Knowledge, skill, and self-efficacy in the secondary prevention of disordered eating behaviors
Sharma et al (2011), India^[42]^	4 wk	Sending text messages with the mobile phone: n = 71	Pamphlet: n = 72	E-learning group: 3.6 ± 0.5 Conventional group: 3.3 ± 0.5	VPI of children and, oral health knowledge, attitude, practice of mothers
Mohebbi et al (2009), Iran^[43]^	6 mo	Phone calls and pamphlet: n = 55	Pamphlet: n = 59 No intervention: n = 63	E-learning group: 12.32 ± 0.47 mo Conventional group: 12.34 ± 0.35 mo No intervention group: 12.33 ± 0.48 mo	DT and DE
Shirmohammadi et al (2022), Iran^[44]^	3 mo	Mobile apps: n = 29	Pamphlet: n = 22	E-learning group: child: 4.6 ± 1.2 mother: 36.4 ± 4.5 Conventional group: child: 4.7 ± 1.2 mother: 34.8 ± 5.3	Modified PI, Modified GI, and oral health knowledge, attitude, practice of mothers
Subburaman et al (2021), India^[45]^	6 mo	Mobile apps: n = 70	No intervention: n = 70	18–20 yr	OHI-S, modified GI, and oral health knowledge, attitude, practice
Wu et al (2022), China^[46]^	12 wk	Mobile apps: n = 22	Oral health education at follow-up points: n = 22	17–29 yr	Al-Anezi and Harradine PI, BMOP, oral health behavior, intention, self- efficacy, action planning, coping planning, outcome expectancies, risk perception, social influences, action control
Lee et al (2023), Korea^[47]^	5 wk	Mobile apps and workbook: n = 22	PowerPoint and workbook twice a week: n = 25 No intervention: n = 26	65 older	O’Leary Index, GI, OHIP-14, GOHAI, togue coating, and oral health knowledge, perception
Divdar et al (2021), Iran^[48]^	8 wk	Sending text messages with the mobile phone (pm): n = 35 Sending text messages with the mobile phone (nm): n = 35	No intervention: n = 34	E-learning group (pm): 26.75 ± 4.77 E-learning group (nm) 27.69 ± 4.4 No intervention group: 27.77 ± 3.94	O’Leary Index, behavioral intention, self- efficacy, and oral health knowledge, attitude, practice

BAS = Behavioral Activation System scales, BIS = Behavioral Inhibition System, BMOP = Bleeding on Marginal Probing Index, DE = the upper central incisors for the number of teeth with enamel caries, DMFT = the number of Decayed, Missing, and filled permanent teeth, DT = the number of decayed teeth, eHEALS = the eHealth Literacy Scale, GBI = Gingival Bleeding Index, GI = Gingival Index, GOHAI = Geriatric Oral Health Assessment Index, ICDAS = International Caries Detection and Assessment System, nm = negative message, nmv = negative message video, OHI = Oral Hygiene Index, OHIP-14 = Oral Health Impact Profile-14, OHI-S = Simplified Oral Hygiene Index, PBI = Papillary Bleeding Index, PI = Plaque Index, pm = positive message, pmv = positive message video, QHI = Plaque Scoring System, SWAL-QoL = Swallowing-related Quality of Life scale, USFR = Unstimulated Salivary Flow Rate, VPI = Visible Plaque Index.

### 3.2. Study period and participant characteristics

The follow-up duration of 19 selected studies varied from 3 weeks to 6 months. Short follow-up studies lasted 3 weeks for the teaching of secondary prevention of disordered eating behaviors.^[41]^ Meanwhile, there were 4 studies with a longer follow-up period of 6 months, which provided instruction on improving dietary patterns,^[17]^ long-term health behaviors,^[40]^ oral health promotion with social media,^[45]^ and preventing caries.^[43]^

The total number of participants at the end follow-up was 3731 and their approximate age ranged from 3 to over 65 years in this qualitative synthesis. This current study was divided into 3 developmental stages (e.g., adulthood, adolescence, and childhood) and analysis in quantitative synthesis.

### 3.3. Characteristics of education methods in the e-learning group

In the e-learning group in 19 studies, 14 studies used mobile phones or smart phones and applications (apps) to provide some text messaging or reminders for oral health education. Additionally, 5 studies used phone calls webinars, videos, and web-based training programs on the internet. Although Al-Ak’hali et al,^[33]^ Mohebbi et al,^[43]^ and Lee et al^[47]^ combined e-learning method with conventional method for oral health education, they could investigate respectively the effect of using e-learning compared to conventional education since both test and control group were given same conventional educational method.

### 3.4. Characteristics of outcomes in quantitative synthesis

This study detected the kinds of oral hygiene indices using the 5 plaque indices and 3 gingival indices in the quantitative synthesis. Besides, the characteristics of the oral heath KAP in each selected study are shown in Table 1.

#### 3.4.1. Plaque index.

Plaque Index (PI) and modified PI implemented in Silness and Löe’s studies^[49,50]^ was used in 3 experiments.^[33,39,44]^ An emphasis of this PI was to assess the thickness of the biofilm at the gingival area.^[51]^ This PI scale ranged from 0 to 3 and the modified PI scale from 0 to 2; 0 for no biofilm, 1 for adhering to the biofilm on the gingival margin area of the tooth surface, 2 for the medium soft accumulation that can be seen directly on the gingival margin area of the tooth surface, and 3 for a large amount of soft accumulation on the gingival margin area of the tooth surface.^[51]^

The Oral Hygiene Index (OHI) implemented by Greene and Vermillion in 1960^[52]^ was used in an experiment.^[21]^ This index aimed to estimate the Debris Index (DI) and/or Calculus Index (CI) of the tooth surface. These indices could calculate DI and CI, respectively, and were considered highly useful. Therefore, Deokar et al cited the DI.^[21]^

The Visible Plaque Index^[53]^ was used in an experiment.^[42]^ The index scores were recommended for recording clearly visible biofilm for mesial, buccal, and lingual tooth surfaces.

Al-Anezi and Harradine PI^[54]^ was used in 2 experiments.^[36,46]^ This PI was reported for orthodontic treatment patients. This index was divided into 4 regions on the bracket, which were medial, distal, gingival, and incisal, on the tooth surface to measure the amount of dental plaque. Each site score ranged from 0 to 3, and summed to obtain a total score of dental plaque coverage.^[54]^

The O’Leary Index^[55]^ was used in an experiment.^[47]^ This index score recorded 4 tooth surfaces (facial, lingual, mesial, distal) on the gingival margin by assessing the presence or absence of dental plaque and calculated the formula as follows: total number of presence surfaces with dental plaque/the number of tooth surfaces × 100 (%). A lower score showed better oral hygiene management regarding dental plaque. Lee et al^[47]^ calculated without multiplying by 100 (%).

#### 3.4.2. Gingival index.

Gingival Index (GI) implemented by Löe and Silness^[56]^ was used in 4 experiments.^[21,33,39,47]^ This index aimed to evaluate the severity of gingival inflammation based on color and bleeding and probe 4 positions on gingival margins.^[57]^ The score range was from 0 to 3.

Bleeding on Marginal Probing Index applied by Van der Weijden et al^[58]^ was used in 2 experiments.^[36,46]^ The gingival margin was probed and assessed for absence and amount of bleeding upon each site within 30 seconds.^[59]^

Modified Gingival Index (GI)^[60]^ was used in an experiment.^[44]^ This index indicated the gingival inflammation by excluding bleeding criteria and this inflammation scores from 0 to 4. Shirmohammadi et al^[44]^ used only the scores of 0 or 1 for assessing oral status of child (0: absence of gingival inflammation; 1: presence of gingival inflammation).

#### 3.4.3. Oral health KAP.

Oral health knowledge indices were used in 5 experiments,^[35,39,42,44,47]^ oral health attitude indices in 3 experiments,^[35,42,44]^ and oral health practice indices in 5 experiments.^[35,36,42,44,46]^ The participants of 3 publications^[35,42,44]^ in quantitative synthesis in oral health attitude were only adults.

### 3.5. Study risk of bias assessment

**Figure 2.**
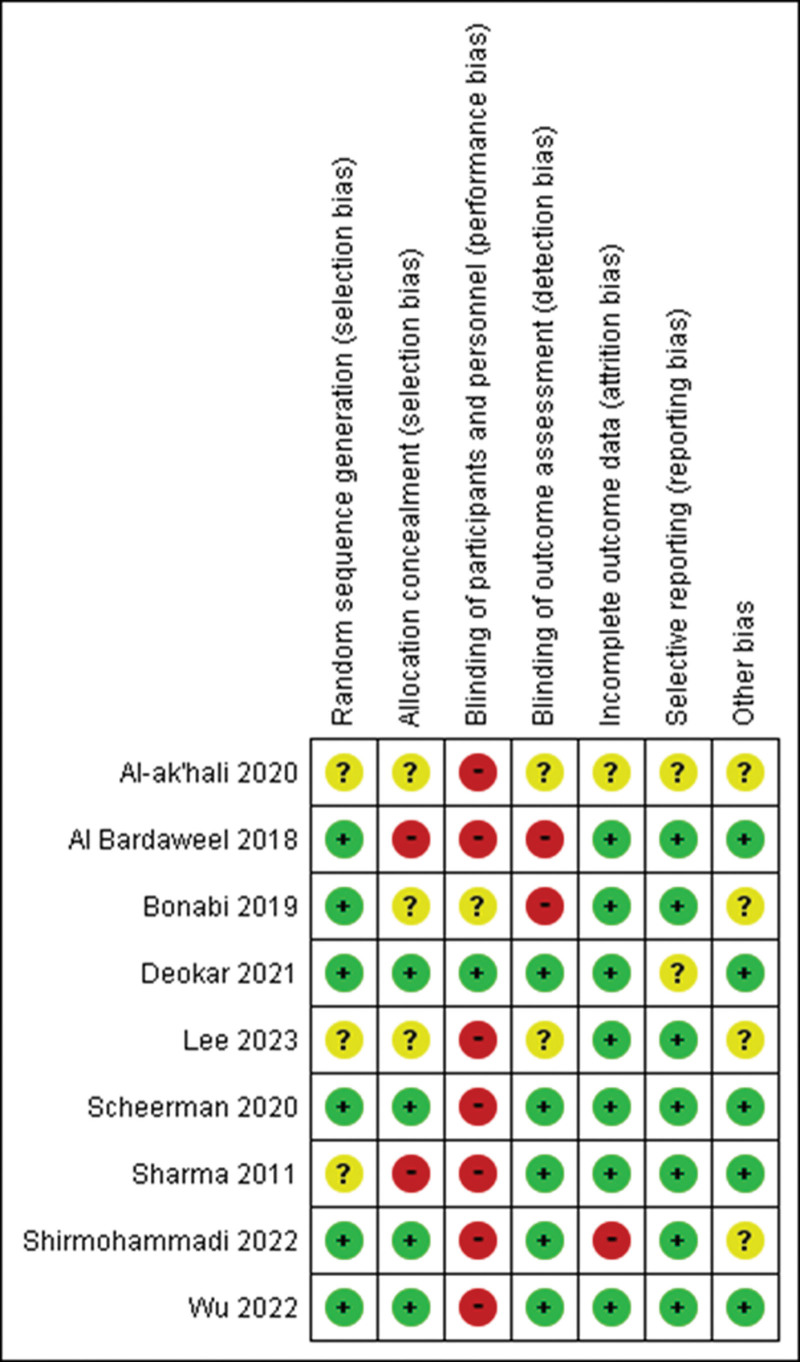
Risk of bias assessment of the RCTs included in the present meta-analysis. RCTs = randomized controlled trials.

The risk of bias graph estimated is shown in Figure 2. All the RCTs collected in the present review have assessed the risk of bias using the Cochrane Handbook for Systematic Reviews of Interventions.^[29]^ In this result of the risk bias summary, the procedures of random sequence generation were implemented in 6 studies,^[21,35,36,39,44,46]^ and 3 studies^[33,42,47]^ were unclear in the present review.

### 3.6. Meta-analysis

**Figure 3.**
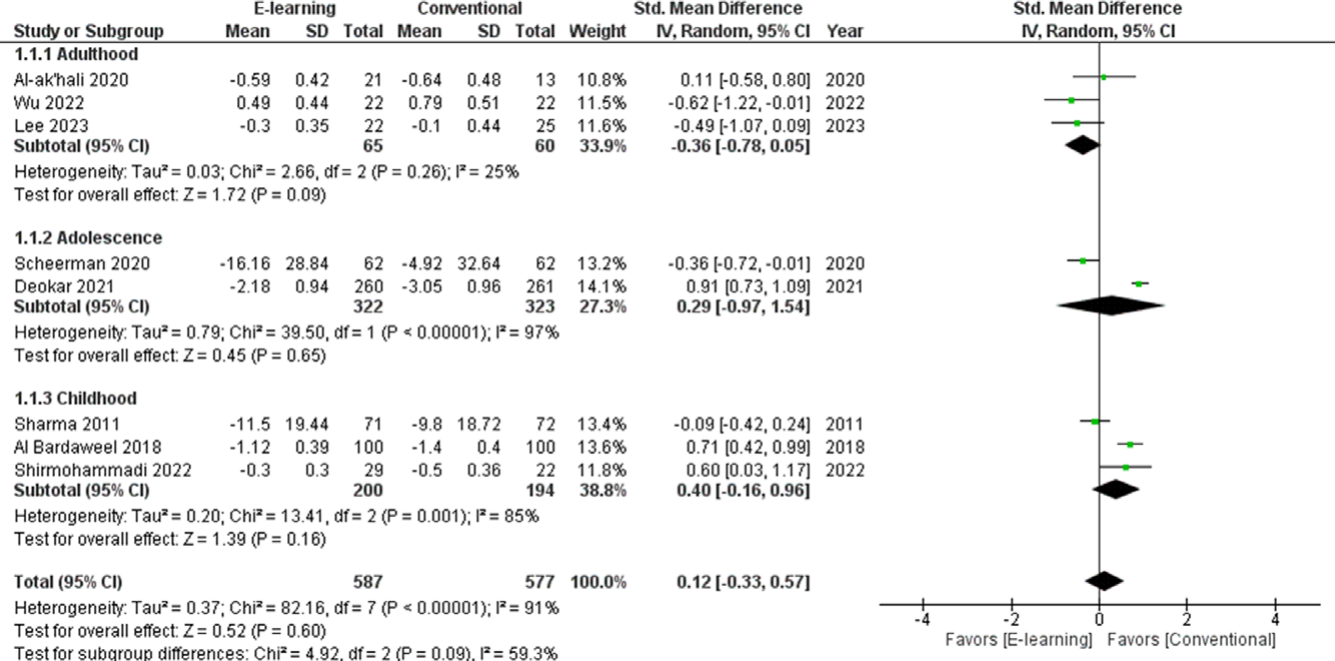
Forest plot of the plaque index scores.

**Figure 4.**
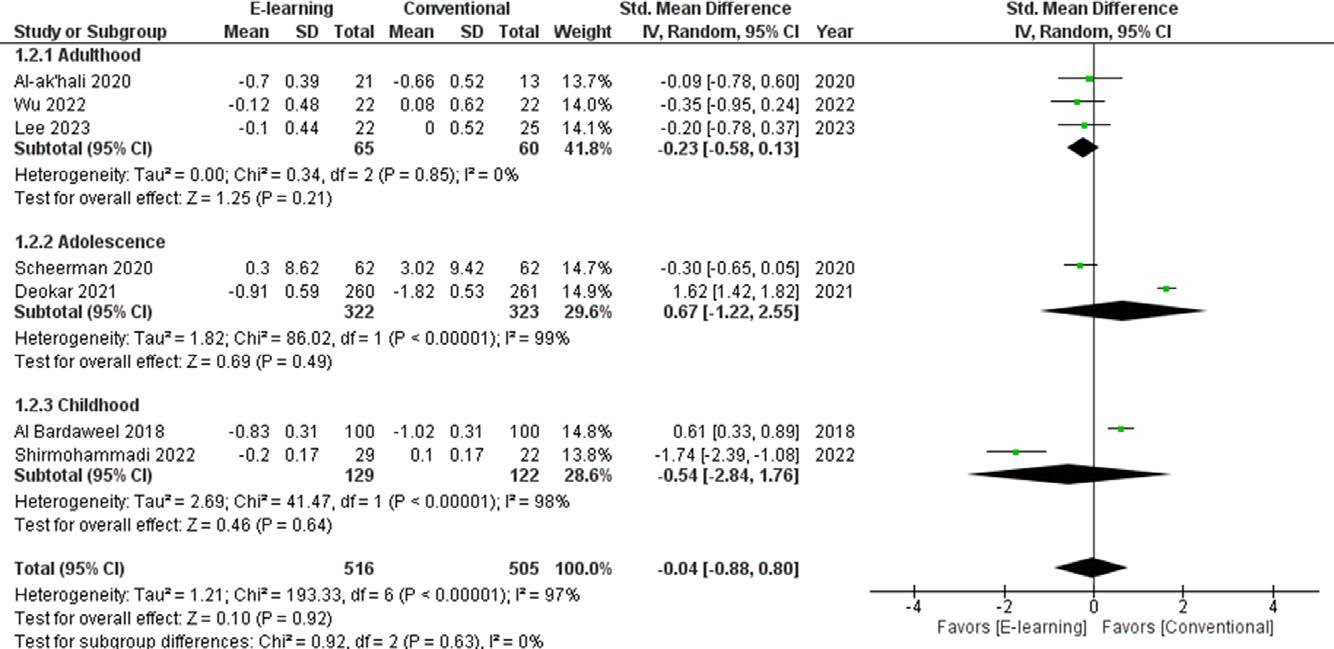
Forest plot of the gingival index scores.

The 9 studies were selected to assess e-learning in comparison with conventional education methods. The pooled standardized mean difference (SMD) of the PI in 8 studies^[21,33,36,39,42,44,46,47]^ was 0.12 (95% CI −0.33 to 0.57) , thus not indicating a better result for the groups that received e-learning (Fig. 3). The pooled SMD of the GI in 7 studies^[21,33,36,39,44,46,47]^ was −0.04 (95% CI −0.88 to 0.80) , thus not indicating a better result for the groups that received e-learning (Fig. 4). High heterogeneity was found for studies regarding both plaque accumulation (I^2^ = 91%, *P* < .001) and gingival inflammation (I^2^ = 97%, *P* < .001). There were no significant differences between e-learning and conventional methods on the oral hygiene state (*P* = .60, *P* = .92, respectively) .

**Figure 5.**
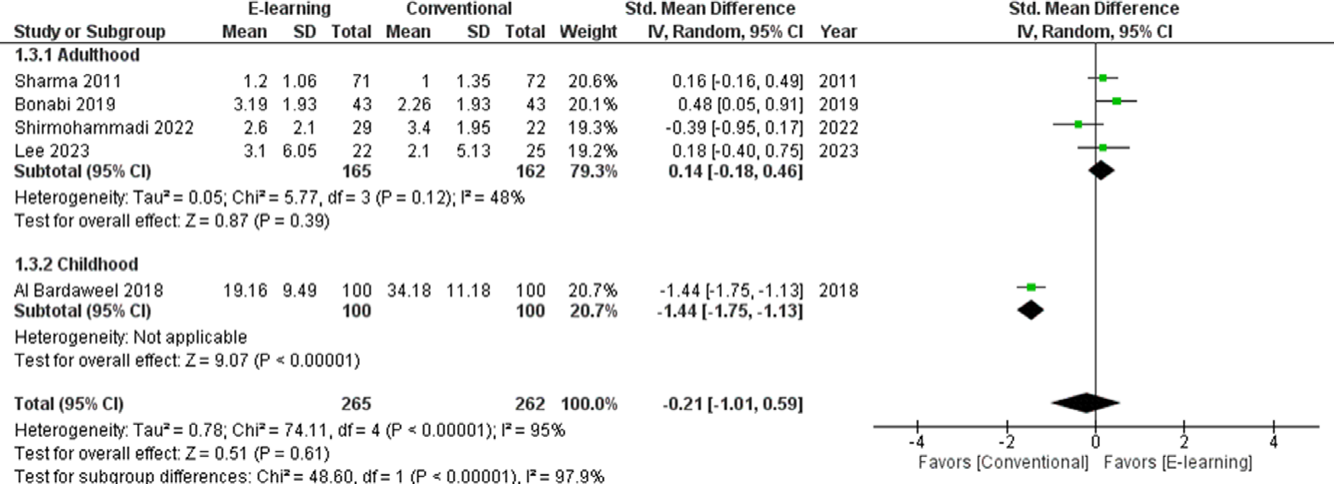
Forest plot of the oral health knowledge scores.

**Figure 6.**
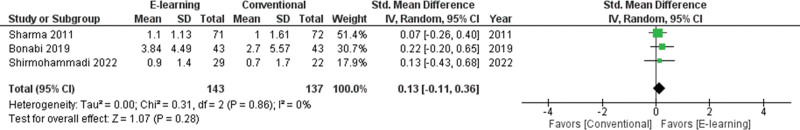
Forest plot of the oral health attitude scores.

**Figure 7.**
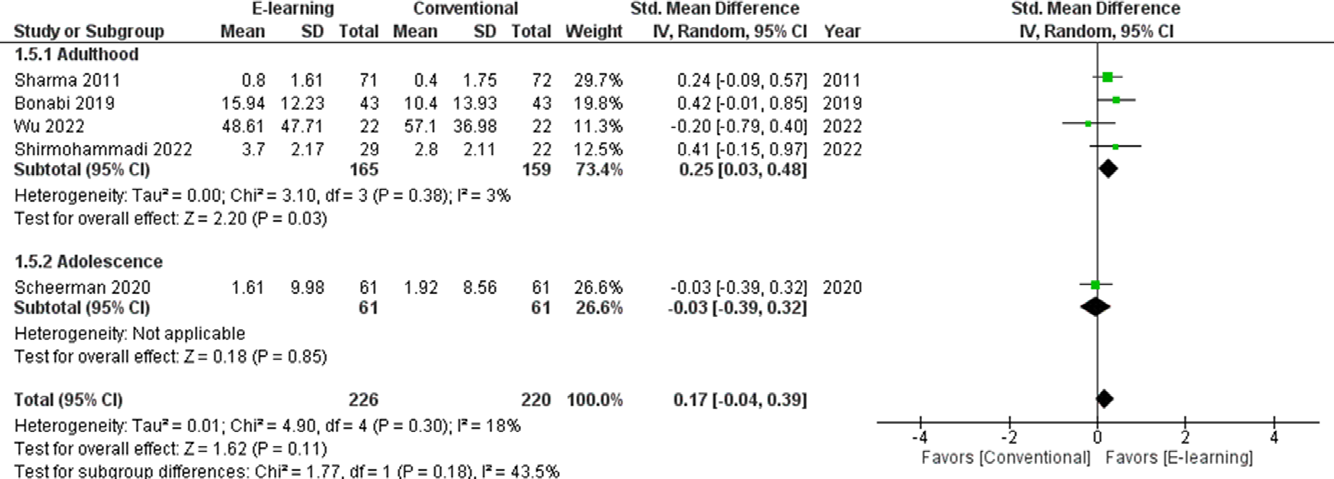
Forest plot of the oral health practice scores.

The subgroup analysis among children for gaining oral health knowledge showed greater effectiveness through conventional education rather than e-learning (SMD = -1.44; 95% CI -1.75 to −1.13; I^2^ = not applicable, *P* < .001; Fig. 5). The subgroup analysis among adults for gaining oral health attitude showed no significant differences between e-learning and conventional education (SMD = 0.13; 95%CI −0.11 to 0.36; *P* = .86; I^2^ = 0%, *P* = .28 ; Fig. 6). The subgroup analysis among adults for oral health practice showed greater effectiveness in terms of e-learning rather than conventional education (SMD = 0.25; 95% CI 0.03 to 0.48; *P* = .38; I^2^ = 3%, *P* = .03 ; Fig. 7).

### 3.7. Reporting biases

The funnel plot to report bias requires 10 studies with the minimum to show the results correctly. Therefore, this meta-analysis did not test the reporting bias because the number of studies was <10 in each result.

### 3.8. Certainty assessment of evidence quality

We assessed the certainty of the evidence of the outcomes included in quantitative synthesis and observes that there is very low and low regarding plaque index, gingival index, oral health knowledge, oral health attitude, and oral health practice (Table 2).

**Table 2:** Summary of the certainty of the outcomes.

Certainty assessment							Certainty
No of studies	Study design	Risk of bias	Inconsistency	Indirectness	Imprecision	Other considerations	
Plaque index 8	Randomized trials	Serious^a^	Serious^b^	Not serious	Serious^c^	None	⨁◯◯◯ Very low
Gingival index 7	Randomized trials	Serious^a^	Serious^b^	Not serious	Serious^c^	None	⨁◯◯◯ Very low
Oral health knowledge 5	Randomized trials	Serious^a^	Serious^b^	Not serious	Serious^c^	None	⨁◯◯◯ Very low
Oral health attitude 3	Randomized trials	Serious^a^	Not serious	Not serious	Serious^c^	None	⨁⨁◯◯ Low
Oral health practice 5	Randomized trials	Serious^a^	Not serious	Not serious	Serious^c^	None	⨁⨁◯◯ Low

a High risk of bias;

b high heterogeneity in meta-analysis;

c not meet the optimal information size criteria.

## 4. Discussion

This systematic review and meta-analysis investigated the question, “Is e-learning more effective in promoting and improving oral health than conventional education?” We conducted a meta-analysis for PI, GI, and KAP related to oral health. Unfortunately, the oral hygiene state indices did not show any positive effect of e-learning in comparison to conventional education in the present study. However, oral health practice with e-learning was more effective for adults than conventional education methods. Similar to our results, some reports have shown that e-learning education increased adults oral health practice scores in randomized controlled trials to date.^[35,44–46,48]^ Since oral health affects and is closely related to systemic health,^[61]^ improving oral health with e-learning may also contribute to general health. In addition, several studies have indicated that e-learning has positively promoted healthy living, such as improving obesity or physical inactivity and inhibiting smoking.^[62,63]^ Our results and the above previous reports may indicate that e-learning education could possibly improve lifestyle habits and prevent lifestyle-related diseases in adults. Furthermore, several studies have shown that medical and dental education has utilized e-learning to provide adults with professional knowledge and skills, such as endodontics,^[26]^ ophthalmology,^[64]^ and dental radiology.^[65]^ Therefore, adults may be able to understand and practice effectively by using e-learning, even if it is specialized education with difficult content. Meanwhile, leaflets such as conventional education were useful for children to obtain oral health knowledge in this study. Reportedly, e-learning education for children regarding oral health knowledge is more effective with game functions,^[66]^ and the importance of clear and interesting e-learning education materials for children has been indicated. Particularly, it is difficult for children, such as preschoolers, to acquire knowledge and practice related to oral health and establish oral hygiene habits themselves because they need parental help.^[44]^ Hence, most of the studies so far have focused on e-learning education aimed at children's parents.^[44]^ For the above results and reports, it seems that e-learning is used easily by adults, while participating in e-learning is difficult for children unless there are enjoyable e-learning functions. However, few studies have compared e-learning and conventional education to prove that hypothesis. Thus, it is necessary to develop enjoyable e-learning education materials for children. In addition, research should be conducted to compare e-learning and conventional education among children or to evaluate the effectiveness of e-learning education compared to conventional education for adults and children in the future.

Additionally, only one study in our meta-analysis results reported on e-learning educational utility for those aged 65 years or older. Lee et al^[47]^ reported a lack of research on e-learning education for older adults thus far, as they are less familiar with mobile devices. Therefore, e-learning that offers oral hygiene education materials aligned with older adults’ cognitive and physical functions should be designed.^[47]^ If user-friendly e-learning materials for older adults are developed in the future, it will become a useful aid to maintain and promote oral and systemic health for those unable to get to the hospital by themselves due to a decline in their physical activity.

The duration of most of the studies selected for meta-analysis in this current study was 3 months. It has been reported that it takes approximately 66 days for people to change their behavior, although the study duration varied depending on the complexity of the behavioral goal to be achieved.^[46]^ Accordingly, a study period of at least 2 months or more is required to encourage participants to improve their behavior using e-learning education. In the present study, most e-learning educational interventions aimed at improving oral hygiene indices and assessing the KAP related to oral health were achieved over 2 months. However, they are unaware whether participants continued using the e-learning and whether e-learning impacted oral health indices after the studies were finished. Shirmohammadi et al^[44]^ reported that e-learning had a more lasting effect than conventional education. Accordingly, it is necessary to prolong the study duration and conduct continuous follow-up after the intervention ends to evaluate the educational effects of e-learning in the future study.

E-learning tools comprise education methods, including e-mail, Microsoft Teams, Skype, Zoom, WhatsApp, and learning management systems such as Moodle.^[67]^

Thus far, e-learning using digital tools has been implemented for oral hygiene to improve plaque removal and gingivitis.^[14]^ Moreover, there have been reports on various kinds of e-learning using the internet.^[68–70]^ Portable devices (i.e., mobile phone, smart phone, and tablet) are useful tools that can provide easy access to information from anywhere. In particular, mobile health, also known as mHealth, uses mobile phone technology to provide health care,^[71]^ Toniazzo et al,^[14]^ Ki et al,^[32]^ and Alkilzy et al^[37]^ indeed reported that mHealth helped in improving oral health indices. Additionally, other experiments reported positive effects of distance learning using the internet.^[72,73]^ However, most control groups in these studies did not include any educational intervention.^[14,17,37,69,72,73]^ Toniazzo et al,^[14]^ Wang et al,^[69]^ and Fernández et al,^[74]^ analyzed the e-learning using mobile health impact on oral health indices and indicated the efficacy of e-learning in systematic review and meta-analysis, whereas those studies were consisted of no education or providing education only at baseline in the control group. Since the educational effect depends on the presence or absence of relevant interventions, control groups should include conventional education methods to examine the effectiveness of e-learning. In this study, we offered a new insight by comparing conventional education methods with e-learning education methods and analyzing the effectiveness of e-learning for different developmental stages. Our interesting results could be used in developing new e-learning education materials and to grow distance learning, like e-learning, in the future.

This study analyzed experiments wherein control groups comprised the provision of conventional education methods. To the best of our knowledge, our findings provided the first evidence for estimating the effectiveness of e-learning in comparison with conventional methods in oral health. Nevertheless, we could not prove the efficacy of e-learning in this meta-analysis, and substantial heterogeneity was detected regarding the oral hygiene state. There are limitations to this study. This meta-analysis included only a small number of experiments. Furthermore, only a few reports investigated the educational effect of e-learning for various developmental stages. Theelen et al reported that studies of e-learning were still in the early stage.^[67]^ Consequently, these were few RCTs that considered the efficacy of e-learning compared with conventional education methods in the field of oral and dental health education until now. In addition, there were only a few studies where the educational contents and frequency for both e-learning and conventional education groups were unified and examined regarding the changes over time in oral hygiene indicators and oral health KAP. Hence, there is a need for well-designed studies involving various age groups to determine whether e-learning for oral health promotion is more effective than conventional education methods.

Our research indicated the positive effect of e-learning education on getting oral health practice among adults rather than conventional education methods for oral health education. However, there were only 9 publications collected in this meta-analysis in total. Therefore, further research should be conducted through high-quality studies on the effectiveness of e-learning in comparison with conventional methods regarding oral health status and KAP in different developmental stages for the future.

## Acknowledgments

The authors thank the Department of Biostatistics, Clinical Research Center in Hiroshima, Hiroshima University Hospital for their valuable advice.

## Author contributions

**Conceptualization:** Yoshino Kaneyasu.

**Data curation:** Yoshino Kaneyasu, Hideo Shigeishi, Kouji Ohta.

**Formal analysis:** Yoshino Kaneyasu.

**Writing – original draft:** Yoshino Kaneyasu.

**Writing – review & editing:** Hideo Shigeishi, Masaru Sugiyama, Kouji Ohta.
